# Identification and Characterization of a Novel Aminoglycoside 3''-Nucleotidyltransferase, ANT(3'')-IId, From *Acinetobacter lwoffii*

**DOI:** 10.3389/fmicb.2021.728216

**Published:** 2021-08-31

**Authors:** Jialei Liang, Kexin Zhou, Qiaoling Li, Xu Dong, Peiyao Zhang, Hongmao Liu, Hailong Lin, Xueya Zhang, Junwan Lu, Xi Lin, Kewei Li, Teng Xu, Hailin Zhang, Qiyu Bao, Mei Zhu, Yunliang Hu, Ping Ren

**Affiliations:** ^1^The Second Affiliated Hospital and Yuying Children’s Hospital, Wenzhou Medical University, Wenzhou, China; ^2^Key Laboratory of Medical Genetics of Zhejiang Province, Key Laboratory of Laboratory Medicine, Ministry of Education, School of Laboratory Medicine and Life Sciences, Wenzhou Medical University, Wenzhou, China; ^3^Institute of Biomedical Informatics, School of Laboratory Medicine and Life Sciences, Wenzhou Medical University, Wenzhou, China; ^4^Institute of Translational Medicine, Baotou Central Hospital, Baotou, China; ^5^Department of Clinical Laboratory, Zhejiang Hospital, Hangzhou, China

**Keywords:** ANT(3")-IId, aminoglycoside-modifying enzyme, aminoglycoside 3"-nucleotidyltranferase, *Acinetobacter lwoffii*, resistance

## Abstract

A novel plasmid-encoded aminoglycoside 3''-nucleotidyltransferase ANT(3")-IId, was discovered in *Acinetobacter lwoffi* strain H7 isolated from a chick on an animal farm in Wenzhou, China. The whole-genome of *A. lwoffii* H7 consisted of one chromosome and five plasmids (pH7-250, pH7-108, pH7-68, pH7-48, and pH7-11). *ant(3")-IId* was identified as being encoded on pH7-250, sharing the highest amino acid identity of 50.64% with a function-known resistance gene, *ant(3")-IIb* (KB849358.1). Susceptibility testing and enzyme kinetic parameter analysis were conducted to determine the function of the aminoglycoside 3"-nucleotidyltransferase. The *ant(3")-IId* gene conferred resistance to spectinomycin and streptomycin [the minimum inhibitory concentration (MIC) levels of both increased 16-fold compared with the control strain]. Consistent with the MIC data, kinetic analysis revealed a narrow substrate profile including spectinomycin and streptomycin, with *K*_cat_/*K*_m_ ratios of 4.99 and 4.45×10^3^M^−1^ S^−1^, respectively. Sequencing analysis revealed that the *ant(3")-IId* gene was associated with insertion sequences (IS) element [ΔIS*Aba14*-ΔIS*Aba14*-hp-orf-orf-orf1-*ant(3")-IId*], and *ant(3")-IId* were identified in plasmids from various *Acinetobacter* species. This study of the novel aminoglycoside 3"-nucleotidyltranferase ANT(3")-IId helps us further understand the functional and sequence characteristics of aminoglycoside 3"-nucleotidyltranferases, highlights the risk of resistance gene transfer among *Acinetobacter* species and suggests that attention should be given to the emergence of new aminoglycoside 3"-nucleotidyltranferase genes.

## Introduction

*Acinetobacter lwoffii* (formerly *Mima polymorpha*, or *Acinetobacter calcoaceticus* var. *lwoffii*) is a Gram-negative aerobic bacillus that inhabits the oropharynx, skin, and perineum (Rathinavelu et al., 2003). As an opportunistic pathogen, *A. lwoffii* can cause infections in patients with impaired or compromised immune systems (Ku et al., 2000; Regalado et al., 2009). Aminoglycosides are highly potent, broad-spectrum antibiotics that act through inhibition of bacterial protein synthesis. Their potent bactericidal activity relies upon binding specifically to the 16S rRNA of the 30S ribosomal subunit, thus interfering with protein synthesis (Mehta and Champney, 2003). There are a large number of aminoglycoside antibiotics, and with the long-term overuse of antimicrobials, multidrug-resistant bacteria, including *A. lwoffii*, have become prevalent worldwide (Mittal et al., 2015). Aminoglycoside resistance mechanisms mainly include methylation of 16S rRNA (Doi et al., 2016), active efflux pumps (Aires et al., 1999), modification of outer membrane permeability or diminished inner membrane transport (Over et al., 2001), and aminoglycoside-modifying enzymes (AMEs; Shaw et al., 1993).

In the clinical setting, resistance to aminoglycosides is most commonly mediated by the presence of various AMEs, including acetyltransferases (AACs), nucleotidyltransferases (ANTs), and phosphotransferases (APHs; Garneau-Tsodikova and Labby, 2016). Among them, ANTs mediate the inactivation of aminoglycosides by catalyzing the transfer of an AMP group from the donor substrate ATP to a hydroxyl group in the aminoglycoside molecule (Ramirez and Tolmasky, 2010). Based on their position specificities for aminoglycoside modification, these enzymes are further divided into several subtypes. There are five classes of ANTs that catalyze adenylation at the 6, 9, 4', 2'' and 3" positions, namely, ANT(6), ANT(9), ANT(4'), ANT(2''), and ANT(3"), respectively (Ramirez and Tolmasky, 2010). The ANT(3") enzymes are the most commonly found ANT enzymes and include two main subclasses (I-II) that specify resistance to spectinomycin and streptomycin. The *ant(3")-I* genes (also known as *aadA* genes) exist as gene cassettes and are part of a large number of integrons, plasmids, and transposons (Parent and Roy, 1992; Naas et al., 1999; Peters et al., 2001). There are more than 20 genes encoding the ANT(3")-I-type enzymes, identified as aadA1 through aadA31 (some numbers are missing). To date, a novel subclass of aminoglycoside 3"-nucleotidyltransferase, ANT(3")-II, has been identified in *Acinetobacter* spp., which comprises numerous variants distributed among three main clades [ANT(3")-IIa, ANT(3")-IIb, and ANT(3")-IIc; Zhang et al., 2017]. The *ant(3")-II* gene not only conferred phenotypic resistance in a given species but was also frequently horizontally transferred between different *Acinetobacter* species (Zhang et al., 2017).

In this work, we identified a novel aminoglycoside 3"-nucleotidyltransferase gene encoding an ANT(3")-II-type enzyme, designated ANT(3")-IId, encoded on a plasmid in *A. lwoffii* isolated from the environment. The function, kinetic parameters and genetic context of the *ant(3")-IId* gene were characterized.

## Materials and Methods

### Bacterial Strains

*Acinetobacter lwoffii* H7 was isolated from an anal swab of a chick from an animal farm in Wenzhou, China. Species identification was initially conducted by the Vitek-60 microorganism auto-analysis system (BioMerieux Corporate, Craponne, France). Further species identification was performed by 16S rRNA gene homology comparison and average nucleotide identity (ANI) analysis using FastANI (Jain et al., 2018). The strains and plasmids used in this work are listed in Table 1.

**Table 1:** Bacteria and plasmids used in this work.

Strain or plasmid	Relevant characteristic(s)	Reference or source
Strain		
H7	The wild-type strain of *Acinetobacter lwoffii* H7	This study
DH5α	*Escherichia coli* DH5α was used as a host for cloning of the *ant(3")-IId* gene	Our laboratory collection
BL21	*Escherichia coli* BL21 was used as a host for expression of the *ant(3")-IId* gene	Our laboratory collection
ATCC 25922	*Escherichia coli* ATCC 25922 was used as a quality control for antimicrobial susceptibility testing	Our laboratory collection
pUCP20-*ant(3")-IId*/DH5α	DH5α carrying the recombinant plasmid pUCP20-*ant(3")-IId*	This study
C600	*Escherichia coli* C600 was used as the recipient in the conjugation experiment, RIF^r^	Our laboratory collection
ATCC 19606	*Acinetobacter baumannii* ATCC 19606 was used as the recipient in the conjugation experiment, AMP^r^	Our laboratory collection
Plasmid		
pUCP20	Cloning vector for the PCR products of the *ant(3")-IId* gene with its upstream promoter region, AMP^r^	Our laboratory collection
pCold I	Expression vector for the PCR products of the ORF of the *ant(3")-IId* gene, AMP^r^	Our laboratory collection

r, resistance; RIF, rifampin; and AMP, ampicillin.

### Antibiotic Susceptibility Testing

The minimum inhibitory concentrations (MICs) were determined using the agar dilution method following the guidelines of the Clinical and Laboratory Standards Institute (CLSI), and the susceptibility patterns were interpreted according to the CLSI breakpoint criteria (CLSI, 2019). The antibiotics tested in this work included 10 aminoglycoside antibiotics (kanamycin, neomycin, ribostamycin, tobramycin, sisomicin, netilmicin, spectinomycin, amikacin, micronomicin, and streptomycin), six β-lactam antibiotics [aztreonam, ceftazidime, cefepime, cefoxitin, meropenem, and ampicillin (AMP)], two quinolone antibiotics (nalidixic acid and ciprofloxacin), two chloramphenicol antibiotics (chloramphenicol and florfenicol), fosfomycin, tetracycline, and polymyxin B. Additionally, as no CLSI breakpoints existed for streptomycin and spectinomycin, so the MIC results for the two antibiotics were interpreted according to the publications by Hu et al. (2017) and Jouybari et al. (2021), respectively. *Escherichia coli* ATCC 25922 was used as a reference strain for quality control.

### Molecular Cloning of the *ant(3")-IId* Gene

The gene encoding ANT(3")-IId was amplified along with its promoter region by PCR with the primers listed in Table 2. The PCR product was digested with BamHI and SphI and ligated into the pUCP20 vector with a T4 DNA ligase cloning kit (Takara Bio, Inc., Dalian, China). The recombinant plasmid was transformed into competent *E. coli* DH5α cells by the calcium chloride method, and the transformant was cultured on Luria-Bertani (LB) agar plates supplemented with 100μg/ml AMP. The size and sequence of the cloned insert was confirmed by restriction enzyme digestion and DNA sequencing.

**Table 2:** Primers used in this study.

Primer^a^	Sequence (5'–3')^b^	Restriction endonuclease	Vector	Annealing temperature (°C)	Amplicon size (bp)
pro-*ant(3")-IId*-F	CGGGATCCTTAATTGTCTATATAGATATTTTAATAAAAACCATGGTC	BamHI	pUCP20	50	1,021
pro-*ant(3")-IId*-R	CATGCATGCTCAGTTAAAAAGTAGTGGTTCAATTTTATG	SphI			
orf-*ant(3")-IId*-F	CCGCTCGAGGACGACGACGACAAGATGCAAAGCTTAAATGATGAAGAGTG	XhoI+EK	pCold I	58	789
orf-*ant(3")-IId*-R	CGCGGATCCTCAGTTAAAAAGTAGTGGTTCAATTTTATGC	BamHI			
q-*ant(3")-IId*-F	TGATGAAGAGTGTCGCCAAG			60	224
q-*ant(3")-IId*-R	TAGCATGACCAATCGGAACA				
H7-16s-F	CAGCTCGTGTCGTGAGATGT			60	151
H7-16s-R	CGTAAGGGCCATGATGACTT				
H7-23s-F	GCAGGTTGAAGGTTGGGTAA			60	174
H7-23s-F	ACAGTGCTCTACCCCCAATG				

a Primers with “orf” were used to clone the ORF of the *ant(3")-IId* gene, primers with “pro” were used to clone the *ant(3")-IId* gene with its promoter region, and primers with “q” were used to perform quantitative real-time PCR (qRT-PCR) analyses.

b The underlined sequences represent the restriction endonuclease sites and their protective bases.

### Quantitative RT-PCR Analyses

To analyze the expression level of ANT(3")-IId, overnight cultures of *A. lwoffii* H7 were diluted in fresh LB with or without supplementation with 1/4 MIC of streptomycin or spectinomycin and grown to mid-log phase. Total RNA was extracted using TRIzol reagent according to the manufacturer’s instructions. RNA purity and concentration were determined spectrophotometrically. DNA-free RNA was confirmed by PCR amplification of the *A. lwoffii* 16S rRNA and 23S rRNA genes. cDNA was synthesized using the PrimeScript RT-PCR Kit (Takara, Dalian, China), and real-time PCR (RT-PCR) was performed using ChamQ Universal SYBR qPCR Master Mix (Vazyme) according to the manufacturer’s protocol. The primers used for quantitative RT-PCR (qRT-PCR) are listed in Table 2. Relative gene expression was calculated using the 2^−ΔΔCT^ method with 16S rRNA and 23S rRNA as the reference genes.

### Plasmid Conjugation Experiment

To detect the transferability of the plasmid pH7-250, *E. coli* C600 (susceptible to rifampicin at >2,048μg/ml) and *Acinetobacter baumannii* ATCC 19606 (susceptible to ampicillin at >128μg/ml) were used as the recipients in conjugation experiments using the filter mating method. The transconjugant was selected on LB plate supplemented with rifampin (RIF; 512μg/ml) or ampicillin (128μg/ml) plus spectinomycin (16mg/ml) or ribostamycin (16mg/ml) and incubated overnight at 37°C. The candidate transconjugant was further analyzed by PCR and sequencing for the presence of resistance genes.

### Expression and Purification of the ANT(3")-IId Enzyme

ANT(3")-IId was overexpressed from *E. coli* BL21(DE3)/pCold I-ANT(3")-IId and purified as described previously with some modifications (Zhang et al., 2017). In detail, the *ant(3")-IId* gene was cloned with an N-terminal His_6_ tag and enterokinase cleavage site into the pCold I vector under the control of the *cspA* promoter using the cold-shock system (Qing et al., 2004). Protein expression was induced with 1mM isopropyl-β-D-thiogalactoside (IPTG) when the culture reached an OD_600_ of 0.6–0.8 at 37°C, and incubation was continued for an additional 16–20h at 16°C. Cells were harvested by centrifugation (5,000×*g*, 10min) at 4°C, resuspended in lysis buffer (20mM Tris-HCl, 150mM NaCl, 3mM β-mercaptoethanol, 0.5% Nonidet-P-40; pH 8.0; Shi et al., 2015), and disrupted by sonication. Cellular debris was removed by centrifugation (10,000×*g*, 30min) at 4°C. The lysates were incubated with pre-equilibrated nickel-nitrilotriacetic acid (Ni-NTA) agarose resin (Beyotime Biotechnology, Shanghai, China) for 8h at 4°C with gentle agitation. The mixture containing the recombinant protein was then loaded onto a column and purified using standard Ni-NTA affinity chromatography. The His_6_ tag was removed by incubation with enterokinase for 3h at 25°C. The digested ANT(3")-IId was purified further using a Ni-NTA column to remove the free His_6_ tag. The purity of ANT(3")-IId was checked by SDS-PAGE, and the protein concentration was determined spectrophotometrically by using a BCA protein assay kit (Thermo Fisher Scientific, Rockford, IL, United States).

### Enzyme Kinetics

The kinetic assay used to monitor activity was performed as reported previously with slight modifications (Kim et al., 2006). The ANT(3")-IId activity was measured by coupling the enzymatic reaction to the reactions of UDP-glucose pyrophosphorylase, phosphoglucomutase, and glucose-6-phosphate dehydrogenase. The catalytic activity of aminoglycoside 3"-nucleotidyltransferase was assayed by monitoring the accumulation of NADPH at 340nm with a Synergy™ Neo2 Multi-Mode Microplate Reader (Biotek, United States). The reaction mixtures contained 50mM HEPES (pH 7.5), 10mM MgCl_2_, 0.2mM UDP-glucose, 0.2mM glucose 1,6-bisphosphate, 0.2mM NADP, 0.2mM dithiothreitol, 2units/ml UDP-glucose pyrophosphorylase, 20units/ml phosphoglucomutase, 20units/ml glucose-6-phosphate dehydrogenase, 1mM ATP, 2μl of ANT(3")-IId, and variable concentrations of aminoglycoside (1–125μM) in a total volume of 0.2ml. Reactions were initiated by addition of the enzyme.

### Genome Sequencing, Assembly, Annotation, and Bioinformatic Analysis

The whole-genomic DNA of *A. lwoffii* H7 was extracted using an AxyPrep Bacterial Genomic DNA Miniprep kit (Axygen Biosciences, Union City, CA, United States). Whole-genome sequencing was achieved using the Illumina HiSeq-2500 and PacBio RS II platforms by Shanghai Personal Biotechnology Co., Ltd. (Shanghai, China). The PacBio long reads were initially assembled by SPAdes v3.14.1 (Bankevich et al., 2012) and Canu v2.1 (Koren et al., 2017). Further correction was conducted by using Pilon (Walker et al., 2014) to improve assembly quality through mapping short reads aligned to the draft of the whole-genome assembly. The ORFs were predicted and annotated using Prokka v1.14.0 (Seemann, 2014) and further annotated by DIAMOND (Buchfink et al., 2015) against the UniProtKB/Swiss-Prot and NCBI nonredundant protein databases with an *e*-value threshold of 1e-5. Annotation of resistance genes was performed using Resistance Gene Identifier (RGI) v4.0.3 in the Comprehensive Antibiotic Resistance Database (CARD; Mcarthur et al., 2013). The molecular weight and pI value of ANT(3")-IId were predicted using ProtParam (Bohm et al., 2020). GView was used to construct basic genomic features (Petkau et al., 2010). Annotation of MGEs was performed using Isfinder (Siguier et al., 2006) and INTEGRALL (Moura et al., 2009). GenoPlotR was used to generate the figure showing structural comparisons and the nucleotide identities between several segments in a linear fashion (Guy et al., 2010). Comparisons of the nucleotide sequences were performed using BLASTN. Multiple sequence alignment and neighbor-joining phylogenetic tree construction were performed using the MAFFT program and MEGAX with a bootstrap value of 1,000 replicates, respectively (Katoh and Standley, 2013; Kumar et al., 2018). Protein sequence motifs of ANT(3")-IId were determined using the MEME Suite.^1^ A predicted amino acid (aa) sequence pairwise identity matrix was generated using MatGAT (Campanella et al., 2003).

### Nucleotide Sequence Accession Numbers

The nucleotide sequences of the chromosome, five plasmids (pH7-250, pH7-108, pH7-68, pH7-48, and pH7-11) of *A. lwoffii* H7 and the *ant(3")-IId* gene reported in this study have been deposited in GenBank under accession numbers CP072549, CP072550, CP072551, CP072552, CP072553, CP072554, and MW984426, respectively.

## Results and Discussion

### Characteristics and the Resistance Profile of *A. lwoffii* H7

The 16S rRNA gene homology analysis suggested that the 16S rRNA gene of H7 had the closest relationship with that of *A. lwoffii* JCM6840 (NR_113346.1, with 95.00% coverage and 99.52% identity), and the ANI analysis revealed that the chromosome sequences of *A. lwoffii* 12CE1 (NZ_CP059081.1) shared the highest identity of 95.71% with H7. Finally, we grouped the strain into the species *A. lwoffii* and named it *A. lwoffii* H7.

The *in vitro* susceptibility test showed that *A. lwoffii* H7 exhibited resistance to eight of the 23 antibiotics tested. It showed higher MIC levels (≥64μg/ml) for ribostamycin, spectinomycin, tetracycline, chloramphenicol, fosfomycin, and nalidixic acid. It was susceptible to many other antibiotics, including aminoglycosides (such as kanamycin, neomycin, and tobramycin) and all the β-lactams tested (except aztreonam; Table 3).

**Table 3:** Minimum inhibitory concentrations (MICs) of 23 antibiotics for five strains (μg/ml).

Antibiotics	ATCC 25922	DH5α	pUCP20/DH5α	pUCP20-*ant(3")-IId*/DH5α	*A. lwoffii* H7
Kanamycin	1	0.25	0.25	0.5	0.25
Neomycin	1	0.25	0.25	0.5	0.25
Ribostamycin	2	2	4	8	128
Tobramycin	0.5	0.25	0.25	0.25	0.25
Sisomicin	0.5	0.25	0.25	0.25	0.25
Netilmicin	0.5	0.25	0.25	0.25	0.25
Spectinomycin	8	8	8	128	64
Streptomycin	2	1	1	16	16
Amikacin	2	1	1	1	4
Micronomicin	0.5	0.25	0.25	0.25	1
Chloramphenicol	4	/	/	/	64
Florfenicol	2	/	/	/	32
Fosfomycin	2	/	/	/	256
Tetracycline	1	/	/	/	64
Aztreonam	0.125	/	/	/	32
Ampicillin	8	/	/	/	16
Ceftazidime	0.0125	/	/	/	8
Cefepime	0.0125	/	/	/	2
Cefoxitin	8	/	/	/	4
Meropenem	0.0125	/	/	/	0.0125
Nalidixic acid	2	/	/	/	128
Ciprofloxacin	0.125	/	/	/	0.5
Polymyxin B	0.125	/	/	/	0.125

### General Features of *A. lwoffii* H7

To better understand the molecular mechanism of drug resistance, the complete genome sequence of H7 was determined. The whole-genome consisted of a chromosome and five plasmids, designated pH7-250, pH7-108, pH7-68, pH7-48, and pH7-11 (Table 4). The chromosome of H7 was 3,040,667 in length, with 43.3% GC content, and encoded 2,805 coding sequences (CDSs). A total of 14 genes with ≥80% similarity with the known antibiotic resistance genes (ARGs) were identified in the whole genome, of which three ARGs were located on the chromosome, 10 and 1 were located on the plasmids pH7-250 and pH7-48, respectively (Supplementary Table S3). When analyzing the resistance mechanism of the isolate against the aminoglycoside antibiotic (spectinomycin), we found an insert sequence (IS) element-related putative *ant(3")-II* gene (finally named *ant(3")-IId* in this work) encoded on plasmid pH7-250 and then cloned it to confirm whether it was functional.

**Table 4:** General features of the *A. lwoffii* H7 genome.

	Chromosome	pH7-250	pH7-108	pH7-68	pH7-48	pH7-11
Size (bp)	3,040,667	250,175	108,848	68,402	48,843	11,166
GC content (%)	43.30	38.30	39.60	37.80	38.00	37.40
Predicted coding sequences (CDSs)	2,805	248	121	76	51	13
Known proteins	2,248	91	83	56	30	9
Hypothetical proteins	557	157	38	20	21	4
Protein coding (%)	76.46	87.43	79.37	78.66	85.20	57.98
Average ORF length (bp)	908	885	718	710	820	501
Average protein length (aa)	309	294	238	236	272	166
tRNAs	86	0	0	0	0	0
rRNA operons	(16S-23S-5S) *7	0	0	0	0	0

### ANT(3")-IId Confers Resistance to Streptomycin and Spectinomycin

Among the resistance genes of known function, *ant(3")-IId* showed the highest aa sequence identity (50.64%) with the aminoglycoside 3"-nucleotidyltransferase ANT(3")-IId (ENU91137.1). Of the 10 aminoglycoside antibiotics tested and compared with the control (*E. coli* DH5α harboring the vector pUCP20 only), the recombinant clone with *ant(3")-IId* [pUCP20-*ant(3")-IId*/*E. coli* DH5α] increased the MIC levels of both streptomycin and spectinomycin by 16-fold, while no significant increase in MIC level was identified for the other aminoglycosides (Table 3).

The qRT-PCR results revealed that *ant(3")-IId* was transcriptionally active under growth in LB free of antibiotics, and treatment with streptomycin or spectinomycin did not result in any significant fluctuation in the mRNA abundance of *ant(3")-IId* (data not shown).

The 3"-nucleotidyltransferase activity and kinetic parameters of ANT(3")-IId against aminoglycosides were in accordance with the MIC results of the cloned *ant(3")-IId*. This enzyme adenylated streptomycin and spectinomycin with *K*_cat_/*K*_m_ ratios of 4.99 and 4.45×10^3^M^−1^ S^−1^, respectively.

### Comparative Analysis of ANT(3")-IId

**Figure 1:**
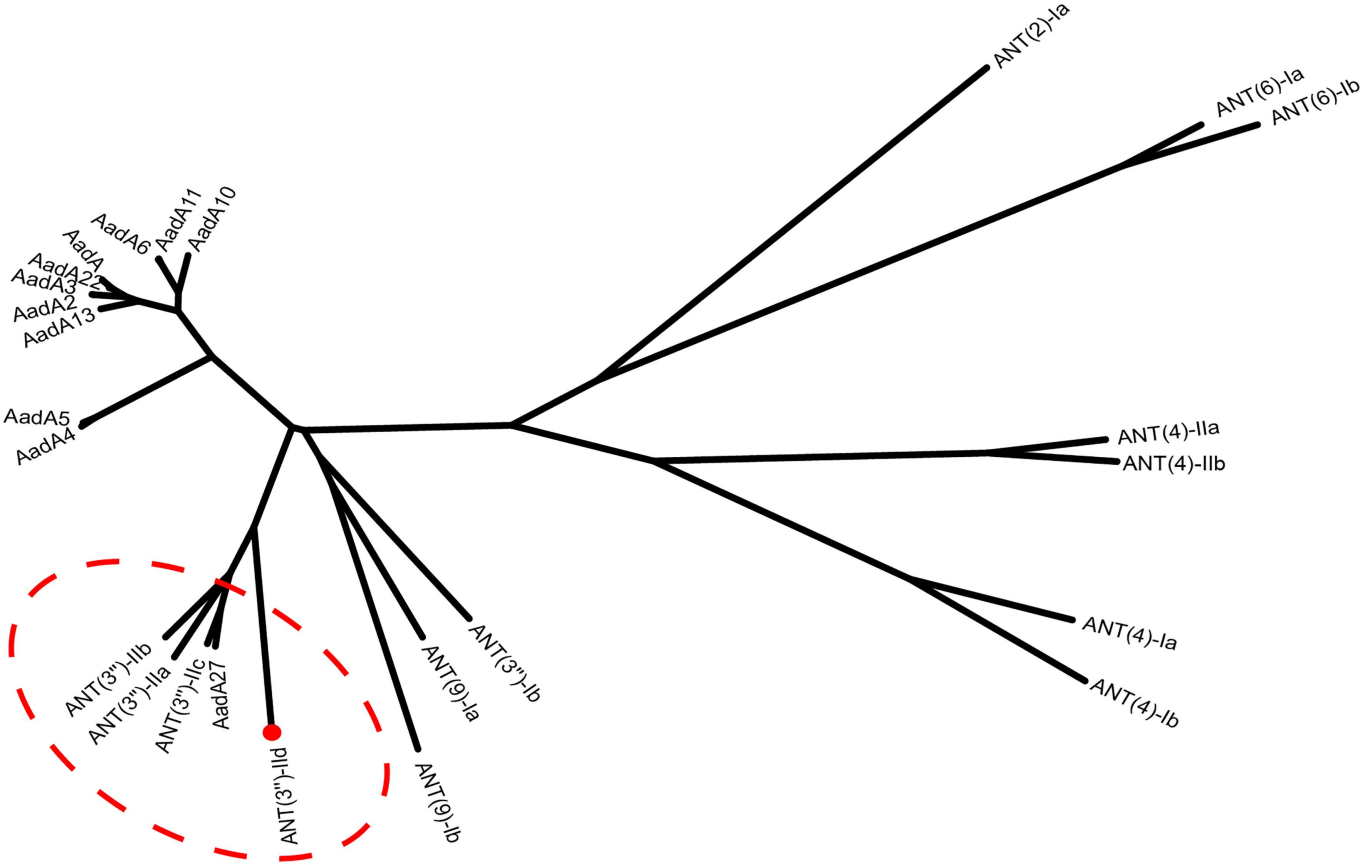
A phylogenetic tree showing the relationship of ANT(3")-IId with other functionally characterized ANTs. ANT(3")-II enzymes are highlighted with a red dashed ellipse. ANT(3")-IId from our study is highlighted with a red filled circle and shown with a red dot.

**Figure 2:**
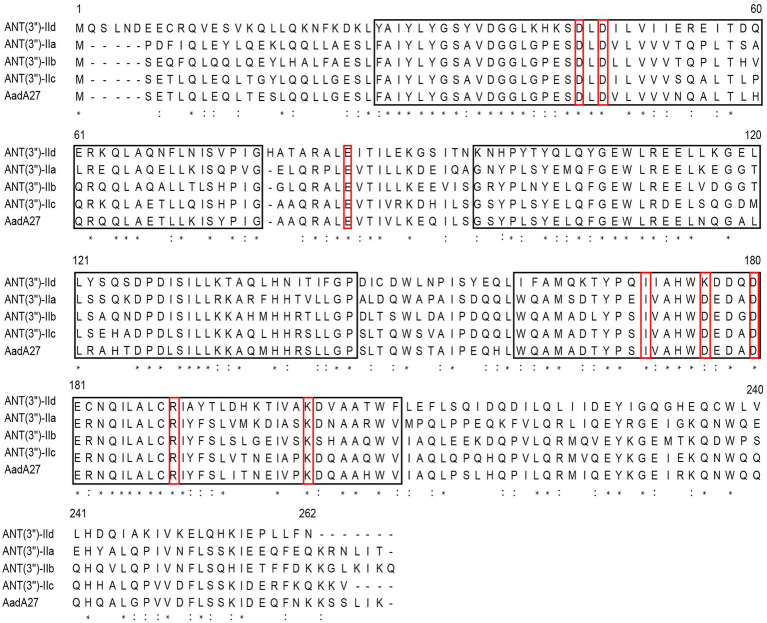
Sequence comparison assay of ANT(3")-II subgroup proteins. The ANT(3")-II proteins and their accession numbers: ANT(3")-IId (this work), ANT(3")-IIa (EEX02086.1), ANT(3")-IIb (ENU91137.1), ANT(3")-IIc (ENU37733.1), and AadA27 (CTQ57092.1). The asterisks indicate fully conserved residues; exclamation marks indicate strongly similar residues. The black frames are the conserved motif sites predicted by the MEME program, and the red frames are some functional positions that have been studied. The numbers correspond to the amino acid residues in each full-length protein.

The *ant(3")-IId* gene is 789bp in length and encodes a 262-amino-acid protein with a molecular mass of 30.38kDa and a pI value of 5.01. It showed an overall identity of 63.7–66.0% with the currently known ANT(3")-II family enzymes. A phylogenetic tree containing ANT(3")-IId and other identified ANTs, including ANT(2'), ANT(4), ANT(6), ANT(9), and ANT(3"), the sequences of which were collected from the GenBank database and previous publications (Shaw et al., 1993; Ramirez and Tolmasky, 2010), revealed that this protein clustered closest to a branch composed of ANT(3")-IIa, ANT(3")-IIb, ANT(3")-IIc, and AadA27 (Figure 1). Sequence comparison assays revealed that this protein shared 50.64, 48.99, 48.40, and 48.00% identity with the four previously reported ANT(3")-II enzymes ANT(3")-IIb, ANT(3")-IIa, AadA27, and ANT(3")-IIc, respectively (Figure 2). Therefore, ANT(3")-IId was assigned as a novel lineage of the ANT(3")-II family.

By searching for homologous genes of *ant(3")-IId* in the NCBI nucleotide database, a total of 15 sequences were retrieved. Notably, these 15 gene sequences were identical to *ant(3")-IId* (100% identity and 100% coverage). Similar to *ant(3")-IId* of *A. lwoffii* from Zhejiang Province, China, these 15 sequences were all from the genus *Acinetobacter*, i.e., *Acinetobacter indicus* (4), *Acinetobacter schindleri* (3), *Acinetobacter towneri* (4), *A. baumannii* (1), *Acinetobacter pisocicola* (1), and (2) from two *Acinetobacter* spp. strains, in addition to *A. lwoffii* H7 from this work. All of them were isolated from four different provinces (Guangdong, Henan, Shanxi, and Jiangsu), China (Supplementary Table S1).

**Figure 3:**
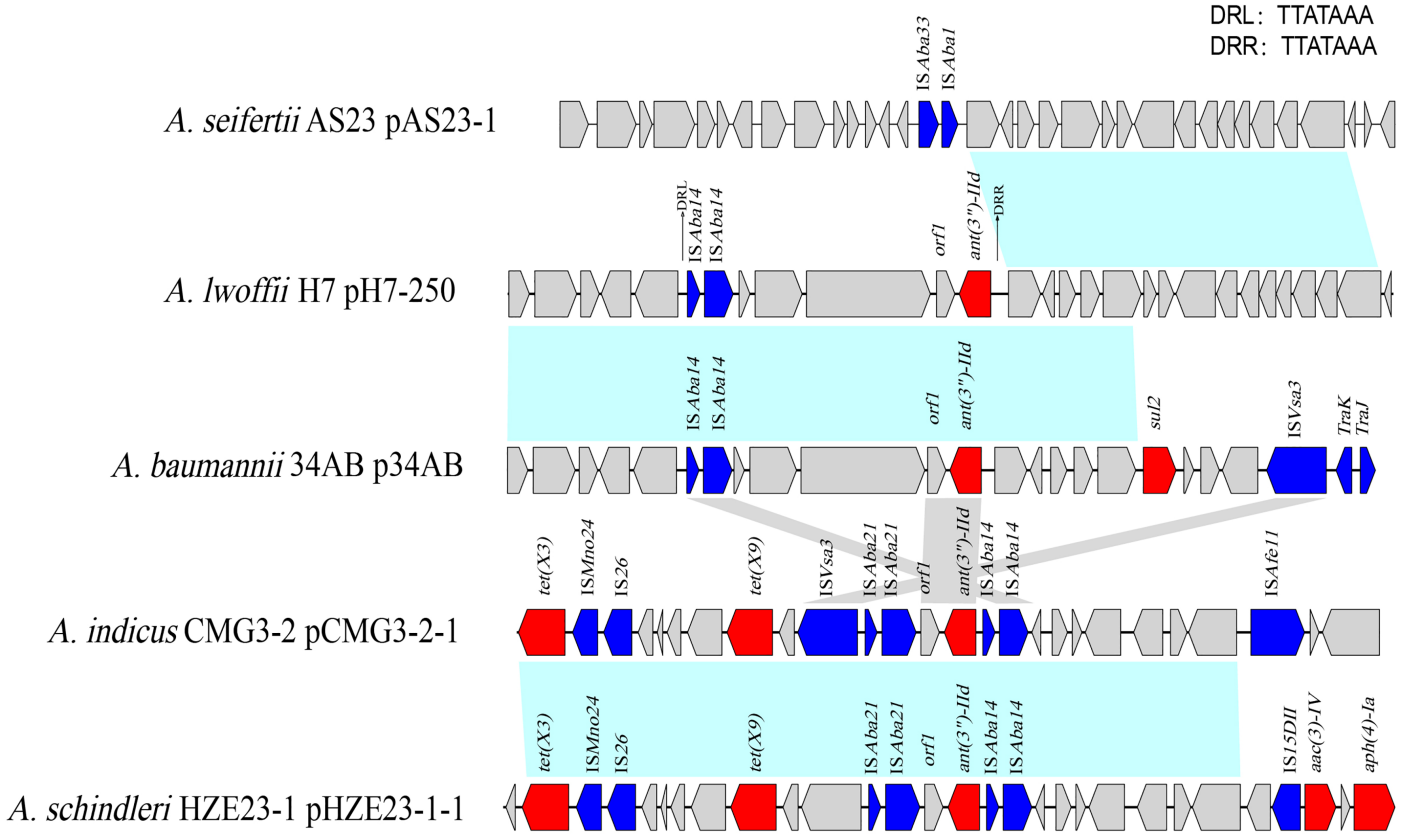
Comparative analysis of the genomic context of the *ant(3")-IId* gene-related region. The *ant(3")-IId* genes in different species were compared. Genes are denoted by arrows and colored according to gene function classification. Blue shading denotes regions of homology (>80% nucleotide sequence identity). The detected direct repeat sequence is marked in the upper right corner. The accession numbers of the sequences are as follows: *Acinetobacter seifertii* plasmid pAS23-1 (CP061673.1), *Acinetobacter baumannii* plasmid p34AB (NZ_MK134375.1), *Acinetobacter indicus* plasmid pCMG3-2-1 (CP044446.1), and *Acinetobacter schindleri* plasmid pHZE23-1-1 (CP044464.1).

Encoded on the plasmids, the *ant(3")-IId* genes were related to the IS elements. Different insert sequences (such as IS*Aba21* and IS*Aba14*) were identified next to or in the proximal region of the *ant(3")-IId* gene. In the upstream region of *ant(3")-IId* of this work were two truncated insert sequences (ΔIS*Aba14*-ΔIS*Aba14*), and the gene array of ΔIS*Aba14*-ΔIS*Aba14*-hp-orf-orf-orf1-*ant(3")-IId* was flanked by a pair of 7-bp perfect direct repeats (DRs), which was characteristic of a typical transposon structure. The different MGEs related to *ant(3")-IId* were identified in the plasmids from various *Acinetobacter* species (Figure 3).

### Comparative Genomic Analysis of the Plasmid pH7-250

**Figure 4:**
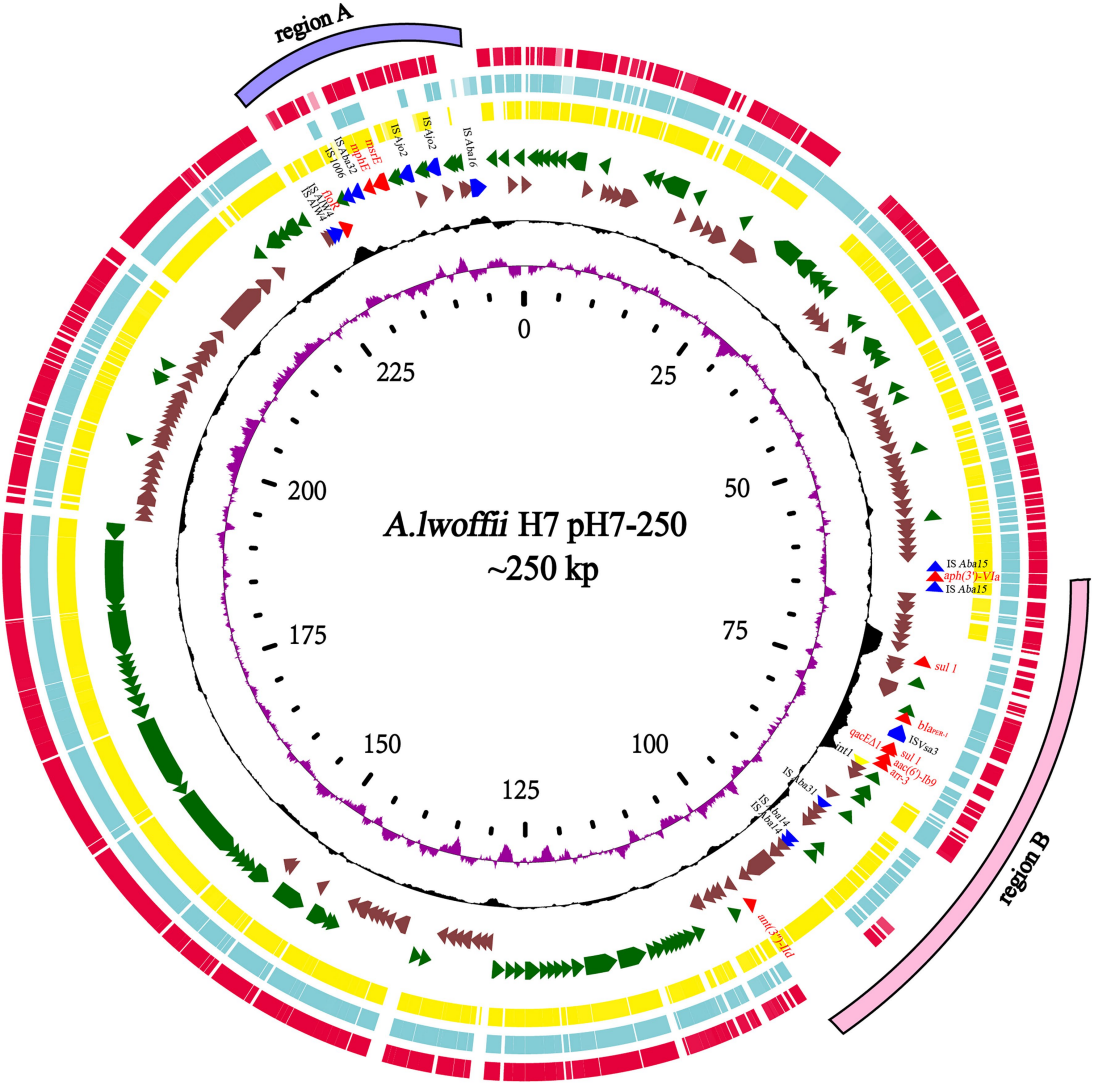
Genomic comparison of the *Acinetobacter lwoffii* H7 plasmid pH7-250 with other plasmids. From outside to inside: circle 1 shows two regions of resistance genes (region A in purple and region B in pink); circles 2, 3, and 4 are homologous regions of the *Acinetobacter haemolyticus* TJR01 plasmid pAHTJR1 (CP038010.1), *Acinetobacter* sp. WCHA55 plasmid pOXA58_010055(NZ_CP032285.1), and *A. baumannii* 34AB plasmid p34AB (NZ_MK134375.1) compared to those of *A. lwoffii* H7, while unmatched regions are left blank; circles 5 and 6 display predicted ORFs encoded in the forward strand and reverse strand, respectively; circles 7 and 8 represent the GC content and GC skew, respectively; and circle 9 shows the scale in kb.

In addition to *ant(3")-IId*, 10 other known resistance genes (similarity ≥80%) were identified as being encoded on the plasmid pH7-250, including two aminoglycoside resistance genes [*aph(3')-VIa* and *aac(6')-Ib9*], one β-lactam resistance gene (*bla_PER-1_*), one chloramphenicol/florfenicol resistance gene (*floR*), one truncated quaternary ammonium compound resistance gene (*qacE*Δ1), two sulfanilamide resistance genes (*sul1*), one rifampicin resistance gene (*arr-3*), and two macrolide resistance genes (*mphE* and *msrE*). These resistance genes were clustered in two regions (designated resistance regions A and B), with *floR*, *mphE*, and *msrE* in one region (7.1kb in size) and the other eight in the other region (39.9kb in size). All the resistance genes were related to MGEs (Figure 4). In resistance region A, *mphE* and *msrE* were surrounded by two inversely oriented p*dif* (XerC-XerD) sites (Blackwell and Hall, 2017), and *floR* was near IS*Alw4*. In resistance region B, as mentioned above, *ant(3")-IId* was related to a transposon, and *aph(3')-VIa* was flanked by a part of the intact insert sequence IS*Aba125*, while the other four resistance genes were carried by a typical class 1 integron with a complete 5'-CS (*intI1*), 3'-CS region (*qacEΔ1*/*sul1*), and variable region [*arr-3*/*aac(6')-Ib9*]. Fifteen plasmid genome sequences with the highest similarities with the pH7-250 genome (>80.0% coverage and >90.0% identity), including p34AB of *A. baumannii* (NZ_MK134375.1, 89% coverage and 99.69% identity), pOXA58_010055 of *Acinetobacter* sp. (NZ_CP032285.1, 93% coverage and 99.81% identity) and pAHTJR1 of *Acinetobacter haemolyticus* (CP038010.1, 91% coverage and 99.73% identity), were retrieved from the NCBI nucleotide database (Figure 4; Supplementary Table S2). Of these plasmid sequences, only p34AB of *A. baumannii* also had the *ant(3")-IId* gene. The complete class 1 integron structure with the variable region [*arr-3*/*aac(6')-Ib9*] on pH7-250 and the MGEs carrying *aph(3')-VIa* were identical to those on pOXA58_010055 and pAHTJR1. The *msrE-mphE dif* module was also found in these three plasmids. Additionally, some type IV secretion system (T4SS) proteins, including DotG/IcmE/VirB10, DotI-like, DotD/TraH, T4SS_TraI, DotA/TraY and TadA, and plasmid-partitioning proteins, including ParM/StbA and ParB/RepB/Spo0J, were predicted in pH7-250. Unfortunately, the plasmid failed to be transferred into recipient cells through conjugation.

## Discussion

In this work, a novel aminoglycoside 3"-nucleotidyltransferase gene designated *ant(3")-IId*, showing the ability to confer resistance to streptomycin and spectinomycin, was identified as being encoded in a plasmid from *A. lwoffii* H7 isolated from chick. ANT(3") enzymes include two main subclasses (I-II) that confer specific resistance to streptomycin and spectinomycin by adenylating the 3'-OH position of the streptomycin glucosamine ring and the 9'-OH position of the spectinomycin actinamine ring (Ramirez and Tolmasky, 2010). In addition to ANT(3")-IId, two other aminoglycoside 3"-nucleotidyltransferases with the highest aa sequence identity with ANT(3")-IId, ANT(3")-IIb (ENU91137.1, 50.64%), and ANT(3")-IIa (EEX02086.1, 48.99%), also showed resistance to the two antibiotics. Compared with the controls, the recombinant carrying *ant(3")-IId* showed 16-fold increased MIC levels for both streptomycin and spectinomycin (from 1 to 16μg/ml and 8 to 128μg/ml, respectively), while both ANT(3")-IIb and ANT(3")-IIa increased the MIC levels (≥64-fold) of both streptomycin and spectinomycin [ANT(3")-IIb, from 4 to 256μg/ml and 32 to 2048μg/ml; ANT(3")-IIa, from 4 to 512μg/ml and 32 to 2048μg/ml, respectively; Zhang et al., 2017].

It has been reported that the four aa residues E87, W112, D182, and 185H/N and the other two residues W173 and D178 of AadA (CAA48215.1, an aminoglycoside adenylyl transferase with a known structural mechanism) were verified to be determinants of spectinomycin and streptomycin resistance, respectively, and this protein shared 34.30% amino acid sequence identity with ANT(3")-IId. These two proteins had the first four residues (E87, W112, D182, and 185H/N) in common but varied in the last two residues (W173I and D178K; Stern et al., 2018). The publication described the construction of the chromosomal mutants W173A and D178A in the *aadA* gene, and the generated strains were subjected to *in vivo* MIC tests with streptomycin and spectinomycin. The MIC values for the two mutants were reduced 10- and 5-fold for streptomycin (from 128 to 12μg/ml and 24μg/ml, respectively) but remained close to the WT MIC values for spectinomycin (from 192 to 192μg/ml and 128μg/ml, respectively; Stern et al., 2018). Therefore, the low resistance levels to streptomycin and spectinomycin and the corresponding low *k*_cat_/*K*_m_ ratio of ANT(3")-IId might be the result of aa residues variations (most likely W173I and D178K) in commonly conserved loci of the functional domains, and this remains to be clarified in future studies.

The *ant(3")-IId* genes identified in this work or found in the other 15 *Acinetobacter* strains were all encoded on the plasmids. None were found to be encoded on the chromosome. When analyzing the plasmid group of these plasmids, to our surprise, no *rep* gene was predicted in any of them. These *ant(3")-IId* genes were all related to the MGEs of the different structures. Except for the one on the plasmid p34AB of *A. baumannii*, which shared the same sequence structure ΔIS*Aba14*-ΔIS*Aba14*-hp-orf-orf-orf1-*ant(3″)-IId* with pH7-250 of *A. lwoffii* H7 in this work, the others all showed different sequence structures from each other. lS*Aba14* and lS*Aba21* both belong to the same IS3 family, and this structure of the left-end extremity of IS*Aba21* and the right-end extremity of IS*Aba14* was first reported in *A. baumannii*, which formed a composite transposon named Tn2114 at the origin of acquisition of *bla_RTG-5_* (Bonnin et al., 2012). Because plasmid-carried resistance genes generally originate from bacterial chromosomes, the origin of this plasmid-encoded resistance gene remained unknown, even though it appeared in different *Acinetobacter* species through horizontal gene transfer.

## Conclusion

In this work, we reported the complete sequence and function of a novel aminoglycoside 3"-nucleotidyltransferase gene, *ant(3")-IId*, present on pH7-250 in *A. lwoffii* H7. The plasmid pH7-250, with an unknown maintenance mechanism, harbors 11 ARGs. The *ant(3")-IId* gene is located inside a composite transposon structure, which has been found in other *Acinetobacter* strains, suggesting a potential threat in the future because of its high horizontal transfer capability.

## Data Availability Statement

The datasets presented in this study can be found in online repositories. The names of the repository/repositories and accession number(s) can be found below: The datasets presented in this study can be found in the GenBank database as GenBank: CP072549, CP072550, CP072551, CP072552, CP072553, and CP072554 for the chromosome and five plasmids (pH7-250, pH7-108, pH7-68, pH7-48, and pH7-11) of *A. lwoffii* H7 genome sequence, GenBank: MW984426 for the *ant(3")-IId* gene.

## Author Contributions

JLL, KZ, QL, PZ, HML, XZ, and HZ collected the strains and performed the experiments. KZ, HLL, JWL, XL, and KL analyzed the experimental results. JLL, XD, and TX performed the bioinformatics analysis. JLL, KZ, and QB wrote the manuscript. MZ, YH, and PR designed the work. All authors contributed to the article and approved the submitted version.

## Conflict of Interest

The authors declare that the research was conducted in the absence of any commercial or financial relationships that could be construed as a potential conflict of interest.

## Publisher’s Note

All claims expressed in this article are solely those of the authors and do not necessarily represent those of their affiliated organizations, or those of the publisher, the editors and the reviewers. Any product that may be evaluated in this article, or claim that may be made by its manufacturer, is not guaranteed or endorsed by the publisher.

## Footnotes

^1^ http://meme-suite.org/
