# Supplementary material for: Identification and Characterization of a Novel Aminoglycoside 3''-Nucleotidyltransferase, ANT(3'')-IId, From Acinetobacter lwoffii
Source: Front Microbiol. 2021 Aug 31;12:728216. doi: 10.3389/fmicb.2021.728216 (PMC8438517; doi:10.3389/fmicb.2021.728216)
Supplement: Supplementary file 1 [file Table_1.DOCX]

**Table S1**. The gene homologs of *ant(3")-IId* in NCBI.

| Strain | Location | Coverage (%) | Identity (%) | Similarity (%) | Accession No. |
| --- | --- | --- | --- | --- | --- |
| *Acinetobacter indicus* CMG3-2 | pCMG3-2-1 | 100 | 100 | 100 | CP044446.1 |
| *Acinetobacter indicus* FS42-2 | pFS42-2-1 | 100 | 100 | 100 | CP046596.1 |
| *Acinetobacter indicus* MMS9-2 | pMMS9-2-1 | 100 | 100 | 100 | CP044451.1 |
| *Acinetobacter indicus* B18 | pB18-2 | 100 | 100 | 100 | CP044457.1 |
| *Acinetobacter schindleri* HZE23-1 | pHZE23-1-1 | 100 | 100 | 100 | CP044464.1 |
| *Acinetobacter schindleri* HZE30-1 | pHZE30-1-1 | 100 | 100 | 100 | CP044484.1 |
| *Acinetobacter schindleri* HZE33-1 | pHZE33-1-1 | 100 | 100 | 100 | CP044475.1 |
| *Acinetobacter pisocicola* YH12207_T | pYH12207-2 | 100 | 100 | 100 | CP046044.1 |
| *Acinetobacter sp* YH12138_T | pYH12138-2 | 100 | 100 | 100 | MK134375.1 |
| *Acinetobacter sp* SH19PTT10 | pYUSHP10-1 | 100 | 100 | 100 | CP048672.1 |
| *Acinetobacter towneri* 19110F47 | p19110F47-2 | 100 | 100 | 100 | CP048661.1 |
| *Acinetobacter baumannii* 34AB | p34AB | 100 | 100 | 100 | MT107270.1 |
| *Acinetobacter towneri* GX7 | pGX7 | 100 | 100 | 100 | CP071772.1 |
| *Acinetobacter towneri GX5* | pGX5 | 100 | 100 | 100 | CP071769.1 |
| *Acinetobacter towneri GX3* | pGX3-1 | 100 | 100 | 100 | CP071767.1 |
