# Supplementary material for: Identification and Characterization of a Novel Aminoglycoside 3''-Nucleotidyltransferase, ANT(3'')-IId, From Acinetobacter lwoffii
Source: Front Microbiol. 2021 Aug 31;12:728216. doi: 10.3389/fmicb.2021.728216 (PMC8438517; doi:10.3389/fmicb.2021.728216)
Supplement: Supplementary file 2 [file Table_2.DOCX]

**Table S2**. The comparison results of pH7-250 genome in NCBI.

| Strain | Plasmid | Coverage (%) | Identity (%) | Accession No. |
| --- | --- | --- | --- | --- |
| *Acinetobacter sp.* WCHA55 | pOXA58_010055 | 93 | 99.81 | NZ_CP032285.1 |
| *Acinetobacter johnsonii* XBB1 | pXBB1-9 | 91 | 99.73 | NZ_CP010351.1 |
| *Acinetobacter haemolyticus* TJR01 | pAHTJR1 | 91 | 98.8 | NZ_CP038010.1 |
| *Acinetobacter ursingii* RIVM0061 | pRIVM0061_IMP-4_171109_B01 | 90 | 99.69 | NZ_MH220287.1 |
| *Acinetobacter defluvii* WCHA30 | pOXA58_010030 | 89 | 99.69 | NZ_CP029396.2 |
| *Acinetobacter pittii* 2014N21-145 | p2014N21-145-1 | 89 | 99.61 | CP033569.1 |
| *Acinetobacter baumannii* 34AB | p34AB | 87 | 99.23 | NZ_MK134375.1 |
| *Acinetobacter baumannii* E47 | pE47_001 | 86 | 99.86 | NZ_CP042557.1 |
| *Acinetobacter pittii* 2014S07-126 | p2014S07-126-1 | 86 | 99.69 | CP033531.1 |
| *Acinetobacter ursingii* RIVM0002 | pRIVM0002_IMP-4_171109_B03 | 86 | 99.46 | NZ_MH220285.1 |
| *Acinetobacter johnsonii* Acsw19 | pAcsw19-2 | 85 | 99.72 | NZ_CP043309.1 |
| *Acinetobacter wuhouensis* WCHAW010062 | pOXA23_010062 | 85 | 99.56 | NZ_CP033130.1 |
| *Acinetobacter baumannii* ABF9692 | pABF9692 | 82 | 99.36 | NZ_CP048828.1 |
| *Acinetobacter ursingii* RIVM0051 | pRIVM0051_IMP-4 | 81 | 99.85 | NZ_MH220286.1 |
| *Acinetobacter pittii* C54 | pC54_001 | 81 | 99.68 | NZ_CP042365.1 |
