# Supplementary material for: Identification and Characterization of a Novel Aminoglycoside 3''-Nucleotidyltransferase, ANT(3'')-IId, From Acinetobacter lwoffii
Source: Front Microbiol. 2021 Aug 31;12:728216. doi: 10.3389/fmicb.2021.728216 (PMC8438517; doi:10.3389/fmicb.2021.728216)
Supplement: Supplementary file 3 [file Table_3.DOCX]

**Table S3**. The details of 14 high similarity ARGs.

| Gene name | Location | Length (bp) | Amino acid Coverage (%) | Amino acid Identity (%) | Amino acid Similarity (%) | GenBank Accession No. |
| --- | --- | --- | --- | --- | --- | --- |
| *bla_oxa-283_* | Chromosome | 831 | 100 | 100 | 100 | WP_004647119.1 |
| *abaQ* | Chromosome | 1311 | 99.08 | 88.43 | 87.62 | ACJ41547.2 |
| *adeJ* | Chromosome | 3117 | 100 | 84.51 | 84.51 | AAX14802.1 |
| *mphE* | pH7-250 | 885 | 100 | 100 | 100 | ABI20451.1 |
| *bla_per-1_* | pH7-250 | 927 | 100 | 100 | 100 | CAA79968.1 |
| *sul1* | pH7-250 | 840 | 90.58 | 100 | 90.58 | AEJ33969.1 |
| *aph(3')-VIa* | pH7-250 | 780 | 100 | 100 | 100 | WP_000422636.1 |
| *msrE* | pH7-250 | 1476 | 100 | 100 | 100 | ACB05808.1 |
| *arr-3* | pH7-250 | 453 | 100 | 100 | 100 | ACD56151.1 |
| *sul1* | pH7-250 | 840 | 90.58 | 100 | 90.58 | AEJ33969.1 |
| *aac(6')-Ib9* | pH7-250 | 588 | 98.46 | 100 | 98.46 | AAD02244.1 |
| *floR* | pH7-250 | 1215 | 100 | 99.26 | 99.26 | AAG16656.1 |
| *qacEΔ1* | pH7-250 | 348 | 100 | 100 | 100 | AAC44316.1 |
| *cmlB1* | pH7-48 | 1266 | 100 | 88.6 | 88.6 | CAL30186.1 |
